# Priming for protection: inducible attachment-resistance to ectoparasitic mites in *Drosophila*

**DOI:** 10.1017/S0031182025100437

**Published:** 2025-08

**Authors:** Ashley L Webster, Michal Polak

**Affiliations:** 1 Department of Biological Sciences, University of Cincinnati, Cincinnati, OH, USA; 2 School of Biological Sciences, The University of Utah, Salt Lake City, UT, USA

**Keywords:** attachment, *Drosophila*, ectoparasites, *Gamasodes* mites, heat shock protein, host defence mechanisms, priming, prophenoloxidase

## Abstract

Ectoparasites are ubiquitous and are often harmful to host fitness. Whereas protective responses to ectoparasitism in vertebrate hosts are well documented, our understanding of such defences in invertebrates remains limited. Here, we examined attachment-resistance in adult *Drosophila* to their naturally co-occurring ectoparasitic mites, *Gamasodes pachysetis* (Parasitidae). Significant differences in mite attachment duration were documented among 6 species of *Drosophila*, providing evidence for interspecific differentiation in attachment-resistance. Experiments with *D. malerkotliana*, a species exhibiting a relatively high rate of mite detachment, revealed that pre-infesting flies significantly reduced mite attachment duration compared to naïve controls, indicating a priming effect. In contrast, a reduction in attachment duration was not observed in *D. malerkotliana* after experimentally wounding the abdominal cuticle. These results suggest that the priming effect is not simply a response to cuticle damage, and that its activation may depend on mite-specific factors. Eight genes were individually tested for their effects on the rate of mite detachment from adult flies by deploying the GAL4-UAS gene knockdown system in *D. melanogaster*. Knockdown of heat shock protein 70Ba (Hsp70Ba) and prophenoloxidase 2 (PPO2), which underlie general stress and melanization responses, respectively, significantly prolonged mite attachment duration, implicating their involvement in host attachment-resistance to mites. Together the results support the existence of inducible protective mechanisms mediating parasitism by mites in a naturally occurring invertebrate host–ectoparasite symbiosis.

## Graphical abstract

**Figure:**
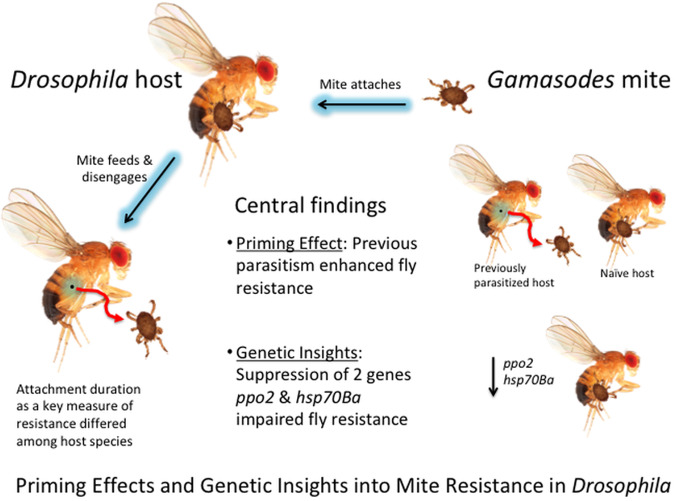

## Introduction

Ectoparasites attack the surface of the host body and play important roles in the ecology and evolution of host species (Behnke, 1990; Clayton et al., 2010; Hopla et al., 1994; Schmid-Hempel, 2021). They infest both wild and domesticated animals, and can generate significant selection pressure in host populations through the damaging effects they exert on host fitness (Lehmann, 1993; Roberts et al., 2013; Wikel and Alarcon-Chaidez, 2001). As a consequence, animals have evolved a wide range of defence adaptations to ectoparasites (Clayton et al., 2010; Hart and Hart, 2018). First-line forms of defence, which are often behavioural in nature, prevent parasite contact and colonization of the body (Nelson et al., 1977), and include host avoidance of infected conspecifics, grooming and preening (de Roode and Lefèvre, 2012; Hart, 1994; Leung et al., 2001; Moyer and Clayton, 2004; Smith, 1988). Second-line defences act from the onset of attachment, and encompass attachment-resistance mechanisms.

Vertebrates are well known to mount second-line defences to a wide range of ectoparasitic arthropods (Allen, 1994; Braden et al., 2020; Brossard and Wikel, 1997; Nelson et al., 1975; Wakelin, 1978; Wilson, 2014). Typically, pronounced immuno-allergic defence responses are elicited by the oral secretions of feeding ectoparasites (Cheng, 1986; Brossard and Wikel, 2008; Owen et al., 2010). Whereas invertebrates are not considered to possess adaptive immunity, they do exhibit highly effective cellular and humoral innate mechanisms to neutralize parasites and pathogens (Schmid-Hempel, 2005; Lemaitre and Hoffmann, 2007; Keehnen et al., 2017). Immune defences of insects have been extensively studied in response to endoparasites, such as the various pathogens and metazoan parasites that invade and develop within the body (Carton et al., 2008; Leitão et al., 2020; Lemaitre and Hoffmann, 2007; Salt, 1970). In *Drosophila*, these responses involve multiple signalling pathways, including the Toll pathway, which are activated upon recognition of pathogen-associated molecular patterns (PAMPs) and mediate both humoral and cellular defences, such as antimicrobial peptide production and haemocyte proliferation (Yu et al., 2022). In contrast, insect host responses to ectoparasites are vastly understudied. The associations between *Drosophila* and their naturally co-occurring ectoparasitic mites are well-suited experimental models for studying insect host–ectoparasite interactions.

Mites commonly infest different species of *Drosophila* that inhabit fermenting fruits and other organic substrates (Campbell and Luong, 2016; Halliday et al., 2005; Perez-Leanos et al., 2017; Polak and Markow, 1995; Yao et al., 2020). Mite species belonging to 2 genera, *Macrocheles* (Macrochelidae) and *Gamasodes* (Parasitidae), are known to breach fly integument with their mouthparts and to feed on host tissue, establishing the parasitic nature of these interactions (Polak, 1996; Polak, M. and Spitz, H., unpublished results). While attached to their hosts, mites use their toothed chelicerae (Halliday et al., 2005; Polak, 1996; Yao et al., 2020) to grasp and pierce the fly’s integument, often producing visible melanized lesions at the feeding site (Polak et al., 2025). Despite this parasitic interaction, *Gamasodes* mites, like *Macrocheles*, are generalist ectoparasites that attack different *Drosophila* species and other insects (Halliday et al., 2005) that co-occur on the same substrate. They are not obligate ectoparasites, however, as they also feed and reproduce on the substrate, where they consume a variety of small invertebrates including nematodes and fly eggs. Mites rely on adult flies to disperse to new habitats (e.g., patches of fermenting fruit) when their current environment deteriorates, becoming dry or otherwise unsuitable for feeding and reproduction. Thus, the interaction between *Drosophila* and *Gamasodes* is shaped both by the mites’ use of flies as dispersal vectors and by their parasitic feeding behaviour during transport.

The fitness consequences of ectoparasitism for flies can be pronounced, with major fitness traits harmed in a dose-dependent manner. For example, parasitism by *Macrocheles subbadius* reduces male mating success (Polak and Markow, 1995; Polak et al., 2007), shortens lifespan (Polak, 1996; Polak and Starmer, 1998), and impairs physiological functions in both sexes (Horn et al., 2020; Polak, 1998). To avoid infestation, *Drosophila* utilize avoidance manoeuvres, bursts of flight from the substrate, and grooming (Polak, 2003; Polak et al., 2023). These first-line forms of defence are heritable, as demonstrated by significant evolutionary responses to artificial selection in the laboratory (Benoit et al., 2025; Luong and Polak, 2007; Polak, 2003; Polak et al., 2023).

Behavioural defences are ineffective once a mite successfully attaches to a host and begins to feed. While host attachment-resistance mechanisms to mites are largely unknown, a recent RNAseq study in *Drosophila melanogaster* provides some insight (Benoit et al., 2020). Infestation by mites triggered differential expression of more than 1300 host genes, of which approximately 900 were overexpressed relative to control, uninfested flies (Benoit et al., 2020). There was transcriptional enrichment of several gene ontology (GO) terms, including immune and stress responses, oxidative stress response, cellular metabolism, plasmatocyte differentiation and melanization. Indeed, host melanin deposition, in the form of a dark-brown, blackish lesion, or ‘scar’, is often directly observed at the mite-induced wound site (Polak et al., 2025). Insect melanotic responses play critical roles in immune defence and wound healing (Cerenius and Söderhäll, 2004; Lemaitre and Hoffmann, 2007) and may interfere with feeding mites (Åbro, 1979; Forbes et al., 1999; Smith, 1988). Additionally, melanin production generates highly reactive, toxic intermediates (Cerenius and Söderhäll, 2004), which could also influence mite feeding duration.

The present study tested the hypothesis that *Drosophila* possesses attachment-resistance mechanisms against *Gamasodes* mites, with the aim of identifying potential mechanisms that may be involved. We first compared mite attachment duration across 6 *Drosophila* species (*D. atripex, D. bipectinata, D. eugracilis, D. malerkotliana, D. melanogaster* and *D. parabipectinata*) known to serve as natural hosts to *G. pachysetis* (Polak et al., 2025; Polak, personal observation). We then tested using *D. malerkotliana* and *D. melanogaster* whether prior mite exposure influenced fly ability to resist subsequent infestation, and whether mechanical injury alone could cause a similar response, as such damage is known to activate pathways involved in haemolymph coagulation and wound healing, among other functions (Nakhleh et al., 2017; Rämet et al., 2002). Finally, we used the GAL4/UAS knockdown system in *D. melanogaster* to examine the potential involvement of 8 candidate genes (Table 1) in mediating mite-attachment resistance, in this case, to *G. queenslandicus*. These genes were selected based on prior transcriptomic data indicating differential expression in response to mite exposure (Benoit et al., 2025) and their annotation with GO terms associated with host responses to mite attachment (Benoit et al., 2020).

**Table 1.** UAS responder lines used in the gene knockdown experiment, 2 control lines and the respective GAL4 line to which UAS responder lines were crossed

FlyBaseID	BDSC stock #	Gene symbol	Gene name	Chromosome
FBgn0015037	67349	*Cypp4p1*	Cytochrome P450 4p1	2
FBgn0004240	67221	*DptA*	Diptericin A	2
FBgn0034407	28975	*DptB*	Diptericin B	3
FBgn0013277	43289	*Hsp70Ba*	Heat shock protein 70Ba	2
FBgn0001233	33947	*Hsp83*	Heat shock protein 83	3
FBgn0035886	51754	*Job66Ci*	Jonah 66Ci	3
FBgn0283437	66938	*PPO1*	Prophenoloxidase 1	2
FBgn0033367	60045	*PPO2*	Prophenoloxidase 2	2
UAS-Control Chr-2	36304	–	–	2
UAS-Control Chr-3	36303	–	–	3
Ubiquitous Gal-4 Driver Chr-2	25374	–	–	2
Ubiquitous Gal-4 Driver Chr-3	3954	–	–	3

FlyBase (https://flybase.org/) ID, Bloomington *Drosophila* Stock Center (BDSC) (https://bdsc.Indiana.Edu/) stock number, gene symbol, gene name and the chromosome on which the gene is located are provided for each gene. All lines were obtained from the Bloomington *Drosophila* Stock Center.

## Materials and methods

### Establishment of Drosophila species cultures

A laboratory culture was established for each of 6 host species: *D. atripex, D. bipectinata, D. eugracilis, D. malerkotliana, D. melanogaster* and *D. parabipectinata*, all of which belong to the subgenus *Sophophora* (Table S1). Voucher specimens of these species are deposited in the Polak Reference Collection at the University of Cincinnati, Ohio, USA.

Each culture was established with approximately 25 field-collected female flies and an equal number of males. Fly species were captured from the surface of fruit substrates within forest or edge habitats, and cultured (see below). Taxonomic information, collection localities, and collection dates for all species are provided in Table S1. All species other than *D. bipectinata* and *D. parabipectinata* were collected between April 20 and May 5, 2022, at different field sites in southwest Thailand, from the vicinity of the town of Ranong (Ranong Province) in the north to Phuket Island (Phuket Province) in the south. *Drosophila bipectinata* and *D. parabipectinata* were collected earlier, in November 2011, in Taipei City, Taiwan. Thus, these 2 species have been maintained in laboratory culture for approximately 130–140 generations longer than the other species, given a laboratory generation time of about 1 month for all species. Once in laboratory culture, no new flies were introduced to the culture of any species, and flies were not exposed to mites during the maintenance of these cultures.

In the laboratory, flies were cultured in 3-4 half-pint glass bottles per generation, and cultures were maintained in an environmental chamber under standard 12 h light (24°C):12 h dark (22°C) conditions. Each new culture generation was seeded with 40–50 flies of each sex per bottle. Culture medium consisted of cornmeal-agar food, prepared using the following base formula: 1800 mL dH_2_O, 100 g yellow cornmeal (Quaker Oats Co., Chicago, IL), 20 g Relax + RF inactive yeast (Lesaffre Corp., Milwaukee, WI), 11 g gracilaria agar (Mooragar Inc., Roseville, CA), 70 mL unsulfured molasses (B&G Foods, Inc., Parsippany, NJ), 18 mL 10% methyl 4-hydroxybenzoate (Sigma-Aldrich, St. Louis, MI) in 95% ethanol and 8.5 mL propionic acid (Thermo Fisher, Waltham, MA).

### Mite cultures

The mite species used in the present work were *Gamasodes pachysetis* Yao and Jin, and *G. queenslandicus* Halliday and Walter, which were originally harvested from bodies of field-caught *Drosophila* at Cape Tribulation, Australia, and in the vicinity of Khao Sok (Surat Thani province), southwest Thailand, respectively. The 2 mite species are morphologically very similar and, to our knowledge, do not differ in behaviour, such as during their interactions with hosts in the laboratory, where both readily attach and parasitize adult flies. Subtle morphological differences, including variation in setal number on the podonotal, opisthonotal and opisthogastric shields, in deutonymphs and adult females, are tabulated in Yao et al. (2020). Both species of mite were cultured in 5-L plastic jugs with a specialized medium (Polak, 2003), and maintained in an environmental chamber at 12-h light (25°C):12-h dark (24°C) conditions.

### Mite detachment times

Flies were experimentally parasitized within infestation chambers containing a culture medium with mites. The infestation chambers are described in Polak (2003). Briefly, each chamber consists of a 300-mL glass jar, approximately one-quarter filled with a mixture of plaster of Paris and activated charcoal powder. The plaster is saturated with water to maintain humidity inside the chamber. Approximately 50–80 mL of mite culture medium containing mites were added to the chamber, and the jar was sealed with breathable mesh. Male flies were aspirated into the chambers in groups of 5–8 through a small perforation in the mesh and continuously monitored for parasitism. Flies that acquired a single mite were immediately aspirated from the chamber and placed individually into 8 fluid-dram polystyrene vials containing cornmeal food. Only flies bearing a single mite attached to the ventral surface of the abdomen, the site on the fly where mites are almost invariably found in both field and laboratory settings. This protocol was used to ensure ecological relevance and in order to avoid potential interaction effects between multiple mites that could influence detachment times. A total of ≈35 parasitized male flies were collected per assay and individually placed into cornmeal food vials, and the vials were housed in an incubator at standard conditions. Parasitized males within vials were observed 3 times daily (9 am, 3 pm, 9 pm) for mite detachments until all flies lost their mite. When a mite was found detached, the detachment time was noted, and the fly was excluded from the assay. Any fly that died before losing its mite was removed from the experiment. Mortality in these assays varied between 4 and 9 deaths over all assays for a given species, ranging from 6.4% for *D. atripex* (*n* = 62) to 12.3% for *D. melanogaster* (*n* = 73).

### Species-level differences in detachment times

We tested for differences in detachment rate of *G. pachysetis* mites across 6 *Drosophila* species. For each host species, flies were reared under density-controlled conditions by allowing sexually mature females to lay eggs for 24 h in culture bottles. Male flies were collected from culture bottles within 6 h of emergence, ensuring that the males were virgin. Virgin males were housed in groups of 15 per vial on standard cornmeal medium under standard incubator conditions for 3–5 days of maturation, after which time they were subjected to experimental parasitism in infestation chambers. Flies parasitized by 1 *G. pachysetis* mite were removed from the chambers and singly transferred to a cornmeal food vial and observed 3 times daily for detachments, as described above. Each fly species was subjected to 2 replicate detachment assays, except *D. melanogaster*, which was tested in 3 replicate assays. To increase the sensitivity of these assays for detecting interspecific differences, we used only male flies in this experiment, as well as in the pre-infection experiment described below. This choice was made to avoid the confounding effects of a potential trade-off between female reproductive investment and immunity, as variation in reproductive effort among females can affect both constitutive and induced immune responses across a wide range of insect species (Schwenke et al., 2016).

The sample sizes for each species were as follows: 62, *D. atripex*; 53, *D. bipectinata*; 54, *D. eugracilis*; 56, *D. malerkotliana*; 73, *D. melanogaster*; and 64, *D. parabipectinata*. Thorax length, used as an estimate of body size (Robertson and Reeve, 1952), was measured under a stereomicroscope using an ocular micrometre in 1 replicate assay of *D. malerkotliana* (*n* = 31) and 1 of *D. melanogaster* (*n* = 34). Flies were frozen in their respective vials on the surface of the culture medium to prevent desiccation and stored frozen for up to 1 week prior to measurement. In these experiments, we used *G. pachysetis* mites because they are naturally associated with this assemblage of fly species studied here and were collected from the same locality (the Khao Sok region of southwest Thailand), providing a biologically relevant pairing for these tests.

### Pre-infestation protocol

To test whether flies that had previously experienced parasitism by *G. pachysetis* mites would exhibit shortened attachment durations in a subsequent occurrence of parasitism, we contrasted attachment times among 3 treatment groups each for *D. malerkotliana* and *D. melanogaster*. Male flies were again only used in these experiments, and were harvested from density-controlled culture bottles and aged in sex-specific food vials for 3–5 days under standard incubator conditions. Male flies were then subjected to the following treatments, generating 3 experimental groups per species: (1) Infested flies were pre-parasitized with 1–2 mites in chambers as described above; (2) exposed control flies were aspirated into chambers with mites but removed before they acquired a mite(s); and (3) unexposed control flies were aspirated into chambers containing the same mite culture medium, but which did not contain mites. All flies remained in the chambers for approximately 60 min. Flies belonging to the different groups were alternately removed from their respective chambers and so were harvested in the same time frame. A total of ≈100 male files were collected for each group. All experimental flies were held in vials with 5–10 males per vial within an incubator at standard conditions for 24 h.

After this 24-h period, all mites were removed from the infested group under light CO_2_ using fine forceps. The presence/absence of mite-induced scars was noted; out of a total of 114 males, 81 were noted to have incurred scars. The unexposed and exposed groups were similarly placed under light CO_2_ and gently touched with forceps. All flies were allowed to recover from anaesthesia for 20 min, after which time they were experimentally parasitized with 1 abdominal mite and individually aspirated into food vials. Flies were parasitized using the same procedure as above to initially infest the parasitized group. A total of ≈40 singly parasitized male flies per group were prepared in this way. All vials were placed in an incubator under standard conditions, and checked for mite detachments as above.

### Cuticle wounding protocol

We tested whether wounding of the host cuticle would subsequently shorten the attachment time by mites. *Drosophila malerkotliana* was used in this experiment because pre-infestation by mites in this species significantly reduced the detachment time of a second mite (see the “Results” section). Post-eclosion, male flies were aged for 3–5 days under standard conditions. Under light CO₂ anaesthesia, the ventral abdominal cuticle of each male was wounded using a sterile minutien dissection pin (1 cm long, 0.018 cm diameter at the tip) by piercing the cuticle to a depth of approximately 0.1 cm. The minutien pin was sterilized between males by dipping it into 70% ethanol and allowing it to air dry. Control males were likewise handled, but their abdominal cuticle was only gently contacted with the pin and not pierced. Two experimental groups were generated, consisting of flies wounded 12 or 24 h prior to infestation. Each experimental group had its matched control group. The time points of 12 and 24 h post-wounding were selected because previous work has shown that flies undergo prominent physiological responses at a wound site at these intervals after mechanical injury with a needle (Rämet et al., 2002).

### GAL4/UAS lines and crosses

To test for the effects of specific genes in mediating mite attachment duration, we used the GAL4/UAS system in *D. melanogaster* to individually suppress 8 genes (Table 1) annotated with host GO terms whose differential transcription has been linked to mite infestation (Benoit et al., 2020, 2025). Gene summaries and biological process annotations for these genes are provided in Table S2. The GAL4 and UAS lines were acquired from the Bloomington *Drosophila* Stock Center (https://bdsc.indiana.edu/), and listed also in Table 1.

All fly lines were cultured in vials with standard cornmeal-agar food under environmental conditions noted above. The crossing protocol involved placing 10–15 virgin UAS-reporter females with 10–15 ubiquitous GAL4-driver males together in food vials and allowing females to reproduce progeny. Each cross consisted of ≈25 vials, and flies were transferred once to fresh food vials after 48 h. Emerging F1 progeny were harvested, and while under a light stream of humidified CO_2_, flies expressing the visible marker specific to the GAL4 line were discarded. In this way, the attachment duration assay used flies carrying the ubiquitous GAL4 driver and UAS responder construct. The control cross was between males from the same GAL4-driver line used in each test cross and virgin females from a ‘UAS-control’ line, which contains all the same transgenic content as the UAS-reporter lines but lacks genomic content downstream of the UAS promoter. The control lines used in the experiment are listed in Table 1. Male and female test progeny from a given line and control cross were aged 4–7 days and then experimentally infested and observed for mite detachments, as above. Both male and female flies were included in these gene knockdown experiments to capture the full range of phenotypic effects associated with gene suppression. Because these experiments tested within-line responses to gene manipulation, including both sexes allowed us to test for any sex-specific consequences of knockdown.

All test lines and the controls were independently assayed for detachment rates in 2 replicates. We used *G. queenslandicus* in the gene knockdown experiments to maintain consistency with previous work, as this species was previously studied in association with stock centre *D. melanogaster* (Benoit et al., 2020).

## Statistical analyses

### Interspecific differences, pre-infestation and cuticle wounding experiments

In these experiments, the response variable was attachment duration, i.e., the time (in hours) between mite attachment and disengagement from its fly host. The factors were species or experimental treatment, and individual male flies the units of replication. Non-parametric Kruskal–Wallis tests compared attachment duration among more than 2 groups, and the Wilcoxon method for pairwise tests (Zar, 2014). Box plots were used to summarize the distribution of the data, presenting medians and interquartile ranges (IQRs). Each box spans from the first quartile (Q1) to the third quartile (Q3), with a line at the median. Whiskers extend to 1.5 times the IQR, and points beyond the whiskers represent potential outliers. Non-parametric tests were used because the raw and transformed data (e.g., square root, log10) failed to meet assumptions of parametric testing. Replicate assays were combined for each species or treatment. The effect of thorax length (an estimate of body size) on attachment duration data was evaluated in *D. malerkotliana* and *D. melanogaster*. These 2 species were selected because they differ significantly in both attachment duration and body size (see the ‘Results’ section), and thus span a range of both variables appropriate for assessing the relationship between them. The relationship between attachment duration and body size for each species was evaluated with the Spearman rank correlation coefficient (*rs*) (Zar, 2014).

Kaplan–Meier survival plots were used to visually represent patterns of detachments over time, and log-rank tests to compare survival distributions (Crawley, 2013). Fits of the data to the Weibull vs the exponential distributions were evaluated using maximum likelihood, and the Weibull was most appropriate (Crawley, 2013).

### GAL4/UAS lines

Mite detachment times for individual flies were measured in males and females from 8 gene knockdown lines. Two groups of lines were analysed separately, consisting of 5 and 3 lines because each of these subsets had its own control line. For each of these 2 groups, an ANOVA was conducted with line, sex, the line-by-sex interaction, and replicate (nested within line), included as factors. Prior to analysis, a square-root transformation was applied to normalize the data; model residuals fit the normal in each analysis on the transformed data (Anderson–Darling test, *P* = 0.12 and 0.06, for the 5- and 3-line models, respectively). A post hoc Dunnett procedure tested each knockdown line mean against a common control (Zar, 2014). For ease of interpretation, means and standard errors were back-transformed to the original scale. The R statistical program 4.2.1 and JMP® Pro 15.0.0 were used for statistical analyses.

## Results

### Species-level differences

**Figure 1.**
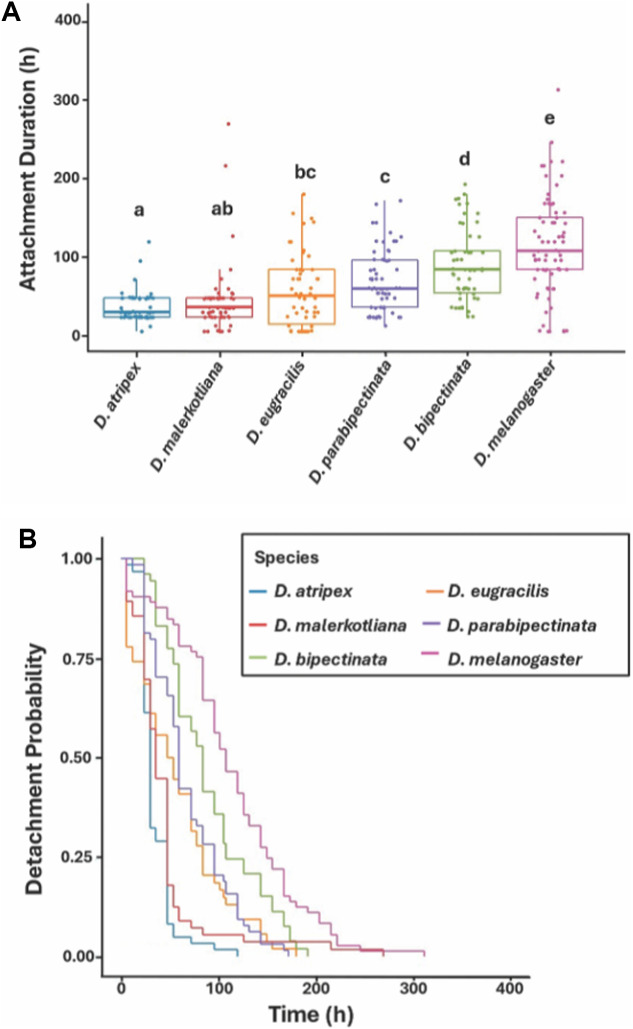
(A) Box plots of attachment duration data for the 6 *Drosophila* species infested by *G. pachysetis* mites (each fly was experimentally infested by a single mite). Medians not sharing a letter are significantly different according to “protected” posthoc Wilcoxon tests (*P* < 0.05). Median (interquartile range, IQR) attachment duration for the different species is as follows: *D. Atripex*: 30 h (24); *D. Malerkotliana*: 36 h (24); *D. Eugracilis*: 54 h (51); *D. Parabipectinata*: 60 h (60); *D. Bipectinata*: 84 h (54); and *D. Melanogaster*: 108 h (66). (B) Survival curves depicting detachment probabilities for the 6 host species.

Median attachment durations of single *G. pachysetis* mites to adult flies differed significantly among the 6 species of *Drosophila* tested (Kruskal–Wallis test, *X*^2^ = 109.823, *df* = 5, *P* < 0.0001). The median attachment durations and IQRs for each species are provided in Figure 1A. This result was supported by congruent differences in the survival curves, which depict detachment probabilities over time (*X*^2^ = 118.6, *df* = 5, *P* < 0.0001; Figure 1B). The species with the shortest median attachment durations were *D. atripex* and *D. malerkotliana*, whereas *D. eugracillis, D. parabipectinata* and *D. bipectinata* exhibited intermediate attachment durations. In contrast, *D. melanogaster* had the longest attachment duration compared to all other species. Relationships between attachment duration and thorax length (estimate of body size) were not significant for either *D. malerkotliana* (*rs* = −0.148, *P* = 0.445) or *D. melanogaster* (*rs* = 0.099, *P* = 0.588). These species differed significantly in mean thorax length (*t* = 21.67, *df* = 1, *P* < 0.0001), with *D. malerkotliana* (mean [s.e.], 0.731 mm [0.00451]) being significantly smaller than *D. melanogaster* (0.866 mm [0.00429]).

### Effect of pre-infestation: D. malerkotliana and D. melanogaster

**Figure 2.**
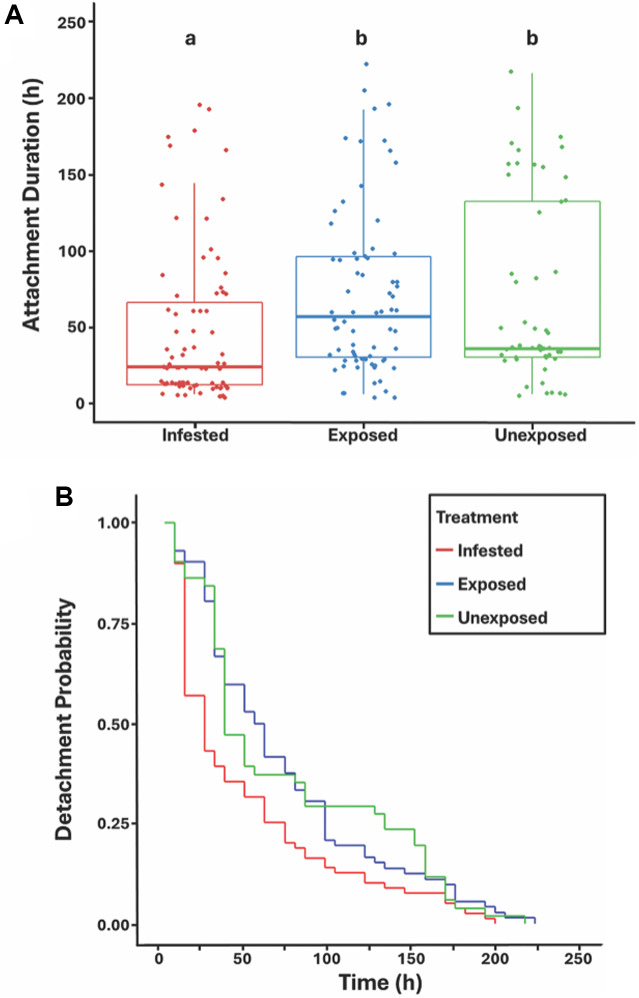
(A) Box plots of attachment duration by *G. pachysetis* mites, for the 3 treatment groups of *D. malerkotliana* in the pre-infestation experiment. Male flies were assigned to 3 groups: infested (pre-parasitized with 1–2 mites), exposed controls (exposed to mites but uninfested), and unexposed controls (exposed to mite-free medium). Medians not sharing a letter are significantly different using post hoc Wilcoxon tests (infested vs Exposed, *P* = 0.0005; infested vs Unexposed, *P* = 0.0005; and exposed vs Unexposed, *P* = 0.839). (B) Survival curves depicting detachment probabilities over time for the 3 treatment groups of *D. Malerkotliana* infested by *G. Pachysetis.*

For *D. malerkotliana*, there was a significant difference among the treatment groups in attachment duration (Kruskal–Wallis test, *X*^2^ = 17.68, *df* = 2, *P* = 0.0005), with the pre-infested group exhibiting a significantly shorter attachment duration compared to both the exposed (by 58%) and unexposed (by 33%) control groups (Figure 2A). Comparison of Kaplan–Meier survival plots confirmed the significant difference in detachment times among the groups (*X*^2^ = 9.56, *df* = 2, *P* = 0.008; Figure 2B). For the pre-infested group, the median attachment duration time was 24 h (24). Median attachment durations for the unexposed and exposed control groups were 36 h (48) and 57 h (42), respectively, and these did not differ significantly from each other (Figure 2A). For *D. melanogaster*, in contrast, the effect of experimental treatment was not significant (Kruskal–Wallis test, *X2* = 3.705, *df* = 2, *P* = 0.157). Similarly, analysis of the survival plots showed non-significant differences (*X*^2^ = 2.99, *df* = 2, *P* = 0.220).

### Cuticle wounding: D. malerkotliana

We tested whether the apparent priming effect detected above for *D. malerkotliana* could be a response to cuticle wounding or a response specific to mite attachment. The median (IQR) attachment duration for flies subjected to wounds 12 h prior to infestation (72 h (72)) did not differ significantly from the control group (72 h (85)) (Wilcoxon test, *W* = 1304.2, *P* = 0.763, *n* = 101). Similarly, median attachment duration for flies wounded 24 h prior to infestation (66 h (70)) did not differ significantly from controls (54 h (48)) (*W* = 1353.5, *P* = 0.418, *n* = 110).

### Gene knockdown

**Figure 3.**
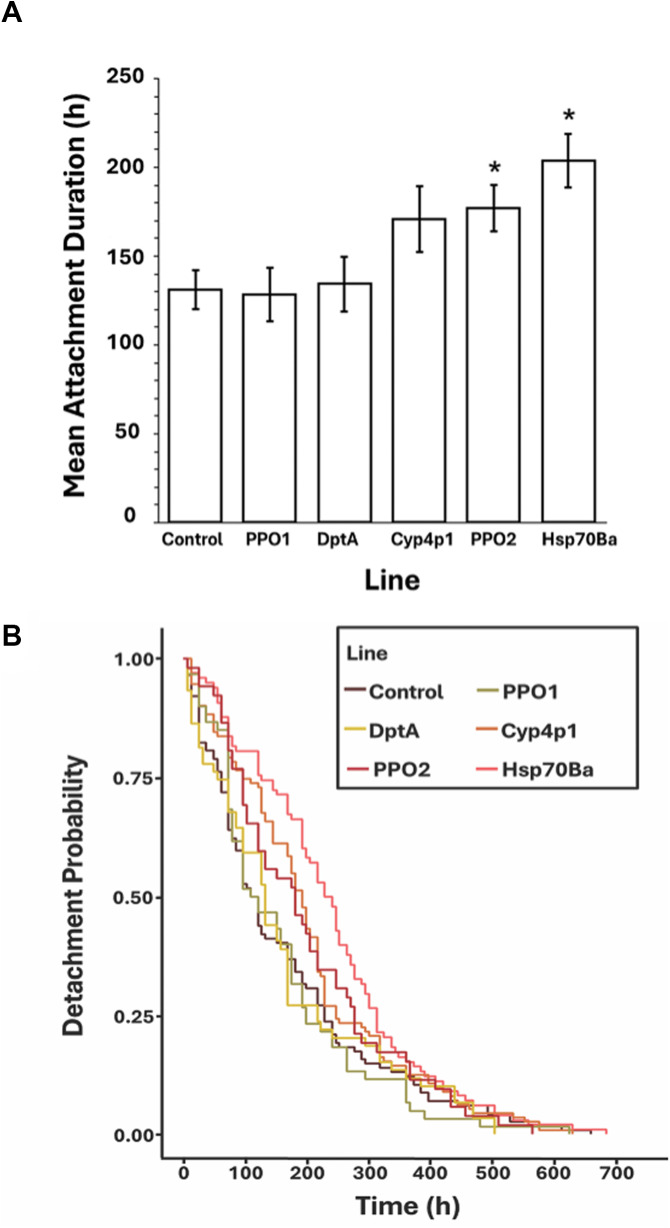
(A) Mean ± SE attachment duration for 5 gene knockdown lines and 1 control line of *D. melanogaster* infested with *G. queenslandicus*. Asterisks indicate the means that differ significantly from the control line by the post hoc Dunnett’s test. (B) Survival curves of detachment probability for the knockdown lines infested with *G. queenslandicus* (*X^2^* = 15.94 *df* = 5, *P* = 0.007).

**Figure 4.**
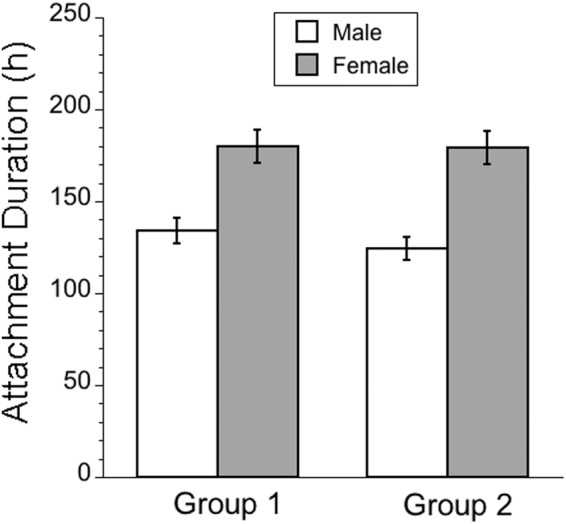
Mean ± SE attachment duration for male and female *D. melanogaster* in the gene knockdown experiment separately for groups 1 (*n* = 6 lines) and 2 (*n* = 4 lines). The effect of sex is significant in each group (*P*s < 0.001; Table 2).

Two ANOVAs were conducted to test for differences in attachment duration, each analysing a distinct subset of gene knockdown (test) lines against a dedicated control line. In a first analysis of 5 knockdown lines, there was a strongly significant overall effect of line (Table 2). A post hoc Dunnett’s test revealed that 2 knockdown lines, heat shock protein 70Ba (Hsp70Ba) and prophenoloxidase 2 (PPO2), exhibited significantly prolonged attachment durations relative to their dedicated control line (*P* = 0.0004 and *P* = 0.0323, respectively; Figure 3A,B). There was also a strong, significant effect of sex on attachment duration, with females exhibiting 34% longer attachment durations than males (Figure 4). There were no significant effects of the line × sex interaction or of replicate. In a second ANOVA on 3 knockdown lines (Table 2), we detected no significant effect of line. However, we again detected a strongly significant effect of sex, with females exhibiting 44% longer attachment duration than males (Figure 4).

**Table 2.** ANOVA results testing for differences in mean attachment duration of mites (*Gamasodes queenslandicus*) among knockdown lines

Source	*df*	SS	*F*	*P*
Group 1^a^				
Line	5	631.07422	4.8370	0.0003
Sex	1	370.53623	14.2003	0.0002
Line × Sex	2	102.54654	0.7860	0.5601
Replicate(Line)	3	177.87012	2.2722	0.0794
Group 2^b^				
Line	3	37.805	0.432	0.730
Sex	1	376.594	12.92	0.0004
Line × Sex	3	24.272	0.278	0.842
Replicate(Line)	2	231.675	3.974	0.020

Two separate analyses were conducted, each focusing on a group of knockdown lines having a specific dedicated control line.

a Group 1: 5 test lines, 1 dedicated control.

b Group 2: 3 test lines, 1 dedicated control.

## Discussion

### Interspecific differences in attachment duration

We documented variation in attachment duration of *G. pachysetis* mites among 6 *Drosophila* species, providing evidence for genetic differentiation in the mite detachment rate from adult flies. To help ensure that any interspecific variation we detected would reflect genetic differences, all host species were reared for multiple generations under standardized laboratory conditions prior to testing. We note, however, that 2 of the 6 species used in our comparison, *D. bipectinata* and *D. parabipectinata*, had been maintained in laboratory culture for approximately 130–140 generations longer than the others. Although all species were reared under the same standardized conditions prior to testing, we recognize that long-term lab culture may lead to differential adaptation (e.g., Matos et al., 2000), potentially contributing to divergence in traits affecting mite detachment. It is also possible that species-specific differences have accumulated over time due to a combination of different evolutionary histories, ecological pressures, as well as prolonged culture in the laboratory. Thus, while our use of uniform laboratory conditions aimed to minimize environmental effects, these conditions differ from the natural environments of wild flies. Consequently, the significant interspecific differences we observed, or their rank order, may not necessarily persist in nature. Numerous factors in natural populations, such as host nutritional history, body condition, stress exposure, age and genotype-by-environment interactions, could interact to potentially alter the pattern of species-specific differences in mite detachment rate that we observed.

Species-specific evolutionary responses to mites or other evolutionary pressures over historic time could explain the differentiation. On the one hand, species may have evolved distinct anti-mite defences, such as structural or semiochemical cuticle properties, or immune responses triggered by PAMPs (Yu et al., 2022), potentially including immunogenic components present on the mites, or introduced by mite saliva or mouthparts during feeding. In odonates, individuals parasitized by *Arrenurus* water mites exhibit variation in haemocyte accumulation, clot formation at wound sites and pronounced melanotic responses induced by mites (Åbro, 1979; Forbes et al., 1999). Alternatively, the differences in the mite detachment rate among species may have resulted from indirect selection arising from variable ecological or climatic stress factors (e.g., other parasites, temperature extremes, UV exposure and desiccation). Indeed, melanin production, known to be critical for immunity in *Drosophila* (Dudzic et al., 2015; Lemaitre and Hoffmann, 2007), also evolves in response to desiccation resistance (Ramniwas et al., 2013). Furthermore, variation in parasitoid encapsulation ability among *Drosophila* species (Kraaijeveld and Godfray, 1997; Ideo et al., 2008; Salazar-Jaramillo et al., 2017) raises the intriguing possibility that immune components differentially shaped by interactions with parasitoids and other parasites (Corby-Harris and Promislow, 2008; Leitão et al., 2020) may have been co-opted, at least partially, for mite defence. Such traits, while not evolved in response to mites *per se*, may nevertheless have rendered certain species physiologically incompatible with mite attachment or feeding, resulting in a form of host–parasite incompatibility driven by other ecological pressures.

Variation in mite attachment duration may also reflect mite preference for host phenotype. Mites likely select hosts based on nutritional properties or dispersal ability cues (Campbell and Luong, 2016), as they rely on hosts to escape deteriorating environments and colonize new habitats (Polak, 1998; Krantz, 1999; Seeman and Walter, 2023). Since *Drosophila* species differ in dispersal ability (Coyne et al., 1982; Markow and Castrezana, 2000), mites may prefer certain species based on cues, such as body size or shape, predictive of dispersal potential. For example, in *Macrocheles muscadomesticae*, host size preference has been documented, with intermediate-sized flies (*D. hydei*) being favoured (Campbell and Luong, 2016). This study, however, assessed preference based on attachment probability rather than attachment duration, limiting its relevance to our results. Our experiments with *D. melanogaster* and *D. malerkotliana* showed no significant relationship between attachment duration and body size within species, suggesting that mite preference based on host size was not an important driver of attachment duration in our study, at least not within the size ranges of these 2 species. The consideration of body size is important, as it could not only provide cues related to host dispersal ability, as noted above, but also influence the duration of mite attachment if variation in host size affects the ability of mites to feed to repletion. Additionally, the lack of a relationship between body size and attachment duration within species also suggests that any variation in host development due to approximately controlled larval density in our study design did not substantially affect the outcome of our assays. These body size results thus make it unlikely that variation in larval density influenced attachment duration.

### Effect of pre-infestation and cuticle wounding

We performed an experiment with 2 species, *D. malerkotliana* and *D. melanogaster*, where previously infested flies were re-infested with mites to test for inducible host defences against mites. We found that in *D. malerkotliana* prior parasitism by a mite enhanced the ability of individual flies to shed a subsequent mite compared to naïve control flies, providing evidence for inducible protective responses. In contrast, this priming effect was not observed in *D. melanogaster*, suggesting that this species either has a weak or absent inducible response specific to the mites, at least under the conditions of our study. Although the reasons for this difference between species are not known, they may reflect different co-evolutionary histories between flies and mites. In particular, species native to the Oriental biogeographic region, such as *D. malerkotliana* (Bock, 1971), which likely have experienced a long-standing evolutionary association with co-occurring *Gamasodes* mites (also of the Oriental region; Halliday et al., 2005), may have evolved effective or specialized defence mechanisms to these mites. By contrast, *D. melanogaster*, a cosmopolitan human commensal originally native to equatorial Africa (Keller, 2007), may have only relatively recently encountered these mites following its expansion into Indo-Pacific habitats, potentially limiting the occurrence of mite-specific defence adaptations in this species.

Previous studies have demonstrated immune priming in various invertebrates (e.g., Bergin et al., 2006; Sadd and Schmid-Hempel, 2006), including *Drosophila* (Pham et al., 2007; Prakash et al., 2024). In general, immune priming (sensitization) enables a faster and stronger response upon subsequent encounters with the same or similar parasite (Sheehan et al., 2020; Schmid-Hempel, 2021). In organisms like insects that lack adaptive immunity, priming can reflect a form of memory within the innate immune system, relying on inducible physiological responses to previous exposure. During priming, organisms exhibit physiological responses to an initial challenge that unfold over a timescale of hours to days, involving dynamic cycles of immune activation and depletion (Schmid-Hempel, 2021). For example, metchnikowin, a *D. melanogaster* antimicrobial peptide with both antibacterial and antifungal activity, is induced after pricking larvae with a bacteria-coated needle, with detectable levels appearing by 6 h post-challenge and peaking at 72 h (Levashina et al., 1998). Additionally, *Drosophila* activate heat shock proteins (Hsps) that peak 1–2 h after heat shock and decline rapidly thereafter (Amstrup et al., 2022; Loeschcke et al., 1997). Notably, the protection from these inducible responses may be felt for days, well after enhanced transcription and peptide production have ceased (Sadd and Schmid-Hempel, 2006).

*Drosophila malerkotliana* in our study displayed an inducible defence pattern to mite challenge consistent with cycles of prominence and depletion. Mite detachments were initially very high after infestation, followed by a slowing of the detachment rate, consistent with a depletion phase. Notably, in flies with a history of prior infestation, the peak detachment period shifted to earlier compared to the 2 control groups, occurring at 20–25 h, with over 50% of mites detached by that time interval (Figure 2b). As detachments progressed, the rate of detachment slowed in all groups. This dynamic shift in the timing of peak detachments characterizes the improved defence response of the pre-infested group, and supports the existence of inducible resistance to ectoparasitism in *D. malerkotliana*.

We investigated whether the observed priming effect in *D. malerkotliana* could be triggered solely by cuticle wounding. However, our results did not support this hypothesis: when the abdomens of *D. malerkotliana* were experimentally wounded with a sterile minutien pin, mite detachment rates did not increase relative to controls. This suggests that other factors, such as mite biting or feeding activity, salivary secretions, or conceivably even tactile stimulation of the fly abdomen, may be necessary, either alone or in concert with wounding, to elicit the priming effect, potentially through the recognition of PAMPs and activation of the Toll signalling pathway (Yu et al., 2022). It is also possible that a single pin insertion did not surpass the threshold of damage required to initiate a robust anti-mite response.

### Gene knock-down effects on attachment by mites

In our survey of 8 candidate genes in *D. melanogaster* for their potential to mediate mite attachment duration, transcriptional suppression of 2 genes, heat shock protein 70Ba (*hsp70Ba*) and prophenoloxidase 2 (*ppo2*), significantly increased attachment duration. Suppression of *hsp70Ba* appeared to exert mildly stronger effects, increasing mean attachment duration by 24.7%, compared to 16.2% for *ppo2*. Hsp70 is a member of the Hsp superfamily, and is a structurally and evolutionarily conserved protein found in both prokaryotes and eukaryotes (Hartl, 1996). In addition to its primary role as a molecular chaperone, which involves promoting the folding and refolding of nascent or stress-denatured proteins, it is also known to regulate homeostasis and sustain anti-infection responses, among other stress-mitigating functions (Gong and Golic, 2006). The remarkable functional breadth of this class of genes suggests a plausible role for Hsp70 also in supporting a defensive reaction to mites. While elucidating the mechanism of gene action lies beyond the scope of our study, it is possible the suppression of *hsp70Ba* disrupted the activity of ≥1 defensive peptides in the haemolymph directly involved in countering feeding mites.

The melanization response, often visible on the fly’s abdomen as darkened patches (scars) at mite feeding sites in *Drosophila* (Polak et al., 2025) is part of the fly’s reaction to mite activity and may serve as a defence against these ectoparasites. Melanin deposition is activated by the Toll pathway and is triggered both systemically following infection and locally in response to cuticle injury (Dudzic et al., 2015; Lemaitre and Hoffmann, 2007). Prophenoloxidase (PPO) catalyses early steps of melanin production and is activated by pattern-recognition proteins that detect microbial compounds or endogenous signals released during tissue damage (Dudzic et al., 2015; Lemaitre and Hoffmann, 2007). In our study, suppressing PPO2 activity may have extended attachment duration of mites by interfering with host melanin deposition or the release of reactive oxidation products (Cerenius and Söderhäll, 2004; Nakhleh et al., 2017). Melanization and its toxic by-products under normal circumstances could restrict mite nutrient intake/processing by interfering with a mite’s feeding apparatus, disrupting enzymatic functions or damaging gut epithelium.

While we did not observe a priming effect in *D. melanogaster*, the significant increase in attachment duration following knockdown of *hsp70Ba* and *ppo2* suggests that these genes could play a role in inducible response to mite attachment in *D. malerkotliana*. One possible explanation for the lack of a priming effect in *D. melanogaster*, despite detecting significant effects on attachment duration via gene suppression, an extreme manipulation, is that parasitism did not induce the appropriate level or combination of specific gene products required for immune priming, as it did in *D. malerkotliana*. It could be that *D. melanogaster*’s immune/stress response systems have been less well optimized by selection for responding to this mite than those of *D. malerkotliana*, leading to the absence of a detectable priming response in *D. melanogaster*. It remains possible, therefore, that *hsp70Ba* and *ppo2* are involved in immune priming in *D. malerkotliana* and other species, identifying a potential avenue for future research.

Finally, it is noteworthy that we observed a sex difference in the ability to shed mites in the gene knockdown experiment, with females overall experiencing significantly longer attachment durations than males. Interestingly, there was no significant interaction between sex and knockdown treatment, indicating that gene suppression affected mite attachment duration uniformly in males and females. The observation that mites consistently exhibited longer attachment durations to female hosts is intriguing because it could reflect an underlying trade-off between female-specific reproductive functions and immune response, both of which require resource allocation (Sheldon and Verhulst, 1996; Ye et al., 2009; Schwenke et al., 2016). There is evidence for such trade-offs in other taxa, where females experience increased susceptibility under conditions of elevated reproductive investment (Gwynn et al., 2005). Alternatively, mites may prefer female hosts because female haemolymph is of superior nutritional quality. A nutritional advantage could explain the evolution of mite preference for female hosts. Future work might focus on discriminating these competing hypotheses and test whether disrupting key female reproductive functions, such as vitellogenin production, affects mite fitness and clearance rate from flies.

## Supporting information

Webster and Polak supplementary material 1 Webster and Polak supplementary material

Webster and Polak supplementary material 2 Webster and Polak supplementary material

## Data Availability

The data are available upon request from the authors.

## Supplementary material

The supplementary material for this article can be found at https://doi.org/10.1017/S0031182025100437.
