# Supplementary material for: Priming for protection: inducible attachment-resistance to ectoparasitic mites in Drosophila
Source: Parasitology. 2025 Jul 10;152(9):897–908. doi: 10.1017/S0031182025100437 (PMC12644947; doi:10.1017/S0031182025100437)
Supplement: Webster and Polak supplementary material 1 — Webster and Polak supplementary material [file S0031182025100437sup001.docx]

**Table S1**. *Drosophila* species used in the interspecific attachment duration experiment, with their taxonomic placements (https://www.taxodros.uzh.ch/) and collection details.

| **Species** | **Subgenus** | **Species group** | **Species subgroup** | **Species complex** | **Collection locality** | | **Collection date** | |  |
| --- | --- | --- | --- | --- | --- | --- | --- | --- | --- |
| *D. atripex* | Sophophora | melanogaster | ananassae | ananassae | | Thailand (Ranong, Phuket Province) | | April - May 2022 | |
| *D. bipectinata* | Sophophora | melanogaster | ananassae | bipectinata | | Taipei City, Taiwan | | November 2011 | |
| *D. eugracilis* | Sophophora | melanogaster | eugracilis | - | | Thailand (Ranong, Phuket Province) | | April - May 2022 | |
| *D. malerkotilana* | Sophophora | melanogaster | ananassae | bipectinata | | Thailand (Ranong, Phuket Province) | | April - May 2022 | |
| *D. melanogaster* | Sophophora | melanogaster | melanogaster | melanogaster | | Thailand (Ranong, Phuket Province) | | April - May 2022 | |
| *D. parabipectinata* | Sophophora | melanogaster | ananassae | bipectinata | | Taipei City, Taiwan | | November 201 | |
