# Supplementary material for: Priming for protection: inducible attachment-resistance to ectoparasitic mites in Drosophila
Source: Parasitology. 2025 Jul 10;152(9):897–908. doi: 10.1017/S0031182025100437 (PMC12644947; doi:10.1017/S0031182025100437)
Supplement: Webster and Polak supplementary material 2 — Webster and Polak supplementary material [file S0031182025100437sup002.docx]

**Table S2**. Functional summaries and biological process annotations for the 8 target genes in the knockdown experiment, sourced from FlyBase (http://flybase.org/) and Gene Ontology Resource (https://geneontology.org/), respectively.

| **Gene symbol** | **Gene summary** | **GO term annotation(s)** |
| --- | --- | --- |
| Cypp4p1 | May be involved in the metabolism of insect hormones and in the breakdown of synthetic insecticides. | Not known |
| DptA | Encodes an immune inducible antibacterial peptide with activity against Gram-negative bacteria. It is expressed in the fat body during the systemic immune response and in various epithelia. Its expression is regulated at the transcriptional level by the immune deficiency pathway. | Response to hyperoxia, response to bacterium, positive regulation of innate immune response, defense response to Gram-negative bacterium, antibacterial humoral response, defense response to bacterium. |
| DptB | Encodes an immune inducible antibacterial peptide with activity against Gram-negative bacteria. It is expressed in the fat body during the systemic immune response and in various epithelia. Its expression is regulated at the transcriptional level by the immune deficiency pathway. | Humoral immune response, response to wounding, defense response to bacterium, defense response to Gram-positive bacterium. |
| Hsp70Ba | Protein involved in response to heat shock and hypoxia. | Response to heat, response to hypoxia, heat shock-mediated polytene chromosome puffing, chaperone cofactor-dependent protein refolding, protein refolding, response to unfolded protein. |
| Hsp83 | A molecular chaperone that helps in the maturation, maintenance, and regulation of specific target proteins. It undergoes a functional cycle that is linked to its ATPase activity, causing conformational changes in client proteins, and leading to their activation. | Positive regulation of insulin receptor signaling pathway, centrosome cycle, negative regulation of cell population proliferation, positive regulation of neuroblast proliferation, cold acclimation, protein folding, proteasome assembly, pole plasm mRNA localization, response to heat, cellular response to heat, membrane bending, oogenesis, multivesicular body fusion to apical plasma membrane, regulation of circadian sleep/wake cycle, sleep, protein stabilization, RISC complex assembly. |
| Job66Ci | Predicted to enable serine-type endopeptidase activity. Predicted to be involved in proteolysis. | Not known |
| PPO1 | Encodes a protein produced by crystal cells (a type of hemocyte cell) and is involved in the melanization reaction, notably upon wounding. | Wound healing, dopamine metabolic process, hemolymph coagulation, response to wounding, defense response to fungus, melanization defense response, scab formation, defense response to Gram-positive bacterium. |
| PPO2 | Encodes a protein stored in large crystals in the crystal cells (a type of hemocyte cell) that is involved in the melanization reaction. It contributes to melanization around wounds and wasp encapsulation. | Melanotic encapsulation of foreign target, dopamine metabolic process, hemolymph coagulation, defense response to fungus, melanization defense response, defense response to Gram-positive bacterium. |
